# Indigestible Substances

**Published:** 1877-06

**Authors:** 


*Indigestible Substances.*—The *Physician* says: The question "How does desiccation convert steamed grains into a wood-like substance?" propounded in the *Times*, by the manufacturers of steam cereals, is easily answered. The gluten, softened by steam, is "fixed" or permanently solidified by drying, and is forever afterward insoluble and indigestible. The same effect is produced upon animal albumen—a kindred substance—and upon gelatine. Modern Chemistry teaches all this, and the experiment is easily tried. The richer the grain treated is in gluten, the more completely insoluble it is rendered. Thus wheat, steamed and dried, is nearly ruined in aroma, flavor, and assimilable nutriment, while oats are less seriously impaired, though rendered insipid and tasteless. The theory that the cereal grains can be in any way improved or their nutritive elements added to by steaming and subsequent drying, is something more serious than a fallacy, since it may induce ignorant persons to employ them as foods, and to suffer by their use.

